# Meta-Analysis of the Effects of Cardiac Rehabilitation on Exercise Tolerance and Cardiac Function in Heart Failure Patients Undergoing Cardiac Resynchronization Therapy

**DOI:** 10.1155/2019/3202838

**Published:** 2019-11-28

**Authors:** Zhang-bing Chen, Liu-bo Fan, Ya-jing Liu, Ya-ru Zheng

**Affiliations:** ^1^Department of Rehabilitation, Taizhou Hospital of Zhejiang Province, Taizhou, Zhejiang, China; ^2^Department of Cardiovascular Medicine, Zhejiang Provincial People's Hospital, People's Hospital of Hangzhou Medical College, Hangzhou, Zhejiang, China

## Abstract

**Objective:**

To evaluate the effects of cardiac rehabilitation on exercise tolerance and cardiac function in heart failure patients undergoing cardiac resynchronization therapy (CRT).

**Methods:**

Randomized controlled trials were initially identified from systematic reviews of the literature about cardiac rehabilitation and heart failure patients with CRT. We undertook updated literature searches of the Cochrane Central Register of Controlled Trials (CENTRAL), PubMed, EMBASE, CBM, CNKI, and Wanfang databases until July 1, 2017. STATA12.0 software was used.

**Results:**

Four randomized controlled studies were included. The total sample size was 157 patients, including 77 in the control group. Cardiac rehabilitation treatment affected the peak VO_2_ in heart failure patients with CRT (*P*_heterogeneity_=0.491, *I*^2^ = 0%). The results lacked heterogeneity, and the data were merged in a fixed-effects model (WMD = 2.17 ml/kg/min, 95% CI (1.42, 2.92), *P* < 0.001). The peak VO_2_ was significantly higher in the cardiac rehabilitation group than in the control group. The sensitivity analysis showed that the results of the meta-analysis were robust. Cardiac rehabilitation treatment affected LVEF in heart failure patients with CRT (*P*_heterogeneity_=0.064, *I*^2^ = 63.6%); the heterogeneity among the various research results meant that the data were merged in a random-effects model (WMD = 4.75%, 95% CI (1.53, 7.97), *P*=0.004). The LVEF was significantly higher in the cardiac rehabilitation group than in the control group. The sources of heterogeneity were analyzed, and it was found that one of the studies was the source of significant heterogeneity. After the elimination of that study, the data were reanalyzed, and the heterogeneity was significantly reduced. There were still significant differences in the WMD and 95% CI.

**Conclusion:**

Cardiac rehabilitation can improve exercise tolerance and cardiac function in heart failure patients with CRT. Future studies are needed to evaluate whether these beneficial effects of cardiac rehabilitation may translate into an improvement in long-term clinical outcomes among these patients.

## 1. Introduction

The incidence of chronic heart failure is increasing annually [1, 2]. In developed countries, the prevalence rate in people over 70 years old is more than 10% [3]. In patients with severe left ventricular systolic heart failure, approximately one-third have ventricular systolic dyssynchrony [4]. In this group of patients, cardiac resynchronization therapy (CRT) can effectively improve exercise tolerance, heart function, quality of life, prognosis, and mortality [5–8]. A large number of clinical studies have shown that cardiac rehabilitation exercise can significantly improve exercise tolerance, cardiac function, quality of life, mortality, and prognosis in patients with heart failure [9, 10]. However, most cardiac rehabilitation studies included only patients with cardiac function classified as NYHA I-II, and few studies were performed on those with cardiac function classified as NYHA II-IV, especially NYHA III-IV. The HF-ACTION study showed that cardiac rehabilitation is safe and effective in NYHA II-IV heart failure patients [11]. Both cardiac rehabilitation exercise and CRT can improve the prognosis of patients with heart failure, but the effect of the combined intervention is still unclear. Due to the small sample size, the inconsistent results, and the lack of systematic evaluation of the quality of the clinical studies, the role of cardiac rehabilitation in patients with heart failure after CRT is still controversial. Therefore, this study aimed at exploring the impact of cardiac rehabilitation therapy on patients with heart failure after CRT and at conducting a systematic, objective, quantitative comprehensive analysis of the existing research results to provide more evidence-based guidance for the treatment of heart failure.

## 2. Materials and Methods

### 2.1. Literature Search Strategy

We searched for randomized controlled trials from January 1, 1990, to July 1, 2017, in the Cochrane Central Register of Controlled Trials (CENTRAL), PubMed, EMBASE, China National Knowledge Infrastructure, Wanfang Data, and China Biology Medicine databases. The keywords used for this search were as follows: (rehabilitation or exercise), cardiac resynchronization therapy, and heart failure. The reference lists of the included studies were also searched for relevant results.

### 2.2. Literature Selection Criteria

**Figure 1:**
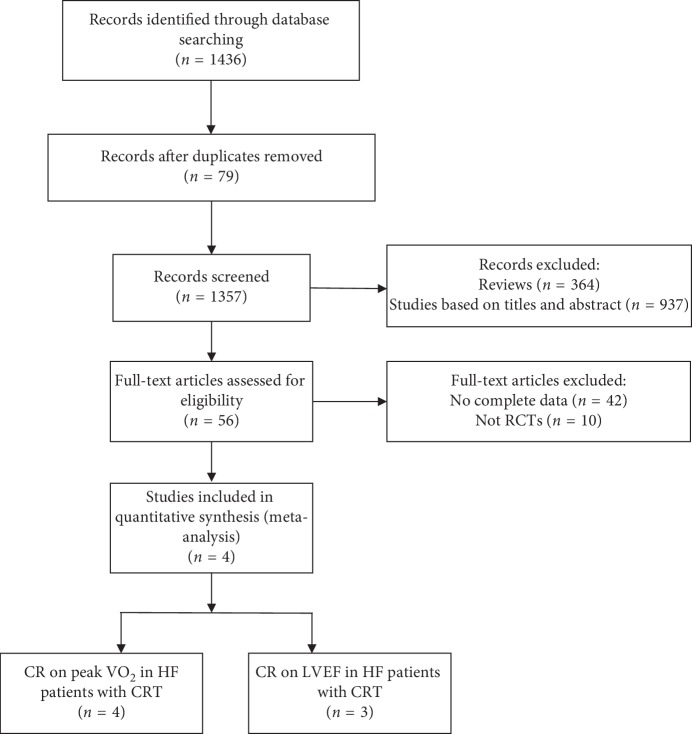
Flow diagram of the study selection procedure used for this meta-analysis of the effects of cardiac rehabilitation on exercise tolerance and cardiac function in heart failure patients with CRT.

Two investigators (Zhang-bing Chen and Ya-jing Liu) independently reviewed all the studies retrieved from the database searches. We followed the guidelines from the Preferred Reporting Items for Systematic Reviews and Meta-Analyses (PRISMA) statement (Figure 1). The selection criteria for this meta-analysis were as follows: (1) studied the effect of cardiac rehabilitation therapy on patients with heart failure after CRT; (2) measured at least peak oxygen uptake (peak VO_2_) or left ventricular ejection fraction (LVEF); and (3) follow-up time less than 6 months. The exclusion criteria were as follows: (1) nonrandomized controlled trial; (2) non-CRT patients with heart failure; (3) interventions that did not include or directly study cardiac rehabilitation and CRT; (4) missing or insufficient original data; (5) repeated reports from the same study; (6) lacking the full text; and (7) follow-up time unclear.

### 2.3. Data Extraction

Two reviewers (Zhang-bing Chen and Ya-jing Liu) extracted the papers independently. We initially reviewed the article titles and abstracts and then excluded those that did not fit the inclusion criteria. All the included studies were extensively reviewed. Any disagreements were resolved by discussion. For each included study, we assessed the methodological rigor and quality using the Cochrane Collaboration's tool for assessing the risk of bias. The information that was extracted included the first author name, year of publication, sample size, follow-up time, LVEF, peak VO_2_, cardiac rehabilitation protocol, and outcome.

### 2.4. Statistical Analysis

All analyses were performed using the STATA software package v12.0 (Stata Corporation, College Station, TX). Pooled effects were estimated by the weighted mean difference (WMD) and a 95% confidence interval (CI) to compare the cardiac rehabilitation group with the control group. We assessed heterogeneity using *χ*^2^-based Q-tests. *I*^2^ values of 25%, 50%, and 75% represented low, medium, and high levels of heterogeneity, respectively. If *P* > 0.05 or *I*^2^ <50%, it was considered that all studies were homogeneous, and a fixed-effects model was selected for the meta-analysis. Otherwise, there was heterogeneity among the studies, and the random-effects model was used in the meta-analysis. Publication bias was assessed using Begg's funnel plot and Egger's linear regression test. *P* values less than 0.05 were considered statistically significant.

## 3. Results

### 3.1. Study Selection

An initial search of the literature yielded 1436 publications. A total of 79 papers had subjects overlapping with other publications. A total of 364 studies were reviews; 937 studies were excluded according to the title and abstract. Then, full-text articles were retrieved and assessed on the basis of the inclusion criteria. Sixty papers were ineligible for the following reasons: 42 papers did not provide complete data for this meta-analysis, and 10 papers were not randomized controlled trials. In the end, 4 studies [12–15] were selected for CR on peak VO_2_ in HF patients with CRT, and 3 studies [12–14] were selected for CR on LVEF in HF patients with CRT. A summary of the study selection process is shown in Figure 1. The characteristics of the included studies are summarized in Table 1.

### 3.2. Effects of Cardiac Rehabilitation on Peak VO_2_ in Heart Failure Patients with CRT

**Figure 2:**
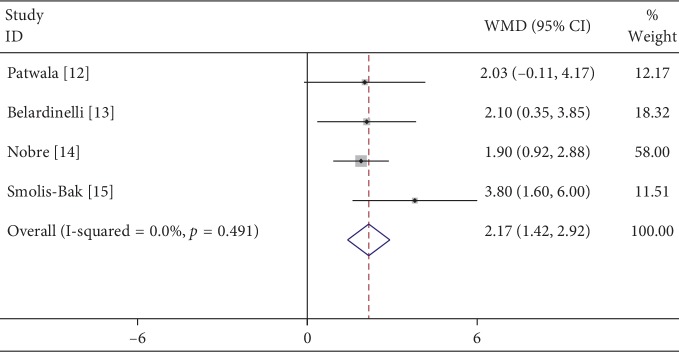
Meta-analysis of the effects of cardiac rehabilitation on peak VO_2_ in heart failure patients with CRT.

A total of 4 studies were included, and the heterogeneity test showed that *P*=0.491 and *I*^2^ = 0%, suggesting homogeneity of all studies. The fixed-effects model was used to merge all data, and the results showed that the peak oxygen uptake of patients in the cardiac rehabilitation group was significantly higher than that in the control group, and the difference was statistically significant (WMD = 2.17 ml/kg/min, 95% CI (1.42, 2.92), *P* < 0.001) (Figure 2).

### 3.3. Effects of Cardiac Rehabilitation on LVEF in Heart Failure Patients with CRT

**Figure 3:**
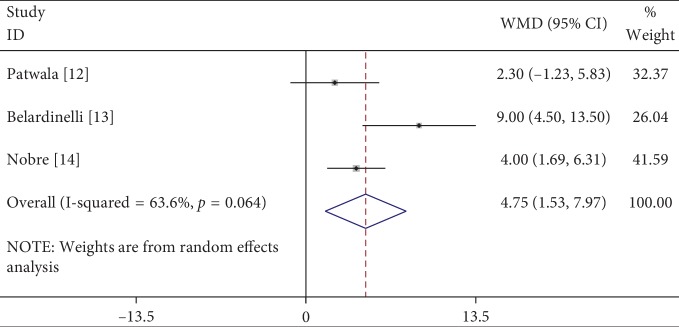
Meta-analysis of the effects of cardiac rehabilitation on LVEF in heart failure patients with CRT.

**Figure 4:**
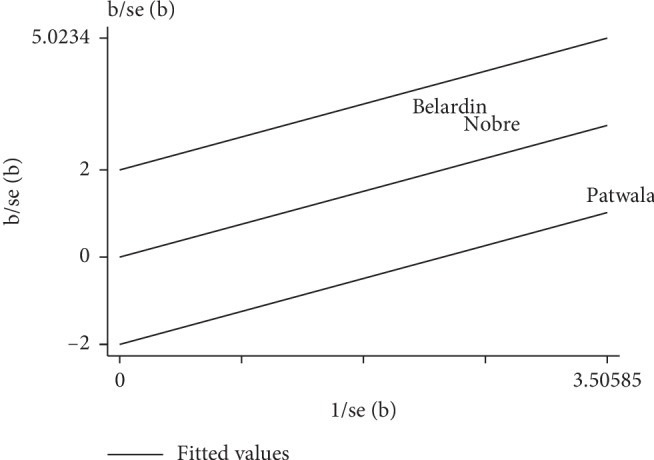
Galbraith plot of the heterogeneity of cardiac rehabilitation on LVEF in heart failure patients with CRT.

A total of 3 studies were included. The heterogeneity test (*P*=0.064, *I*^2^ = 63.6%) suggested that heterogeneity existed in the studies. Data were combined using the random-effects model, and the results were as follows: WMD = 4.75%, 95% CI (1.53, 7.97), *P*=0.004, indicating that patients in the cardiac rehabilitation group had a significantly higher LVEF than those in the control group, and the difference was statistically significant (Figure 3). The Galbraith plot showed the sources of heterogeneity, and there was obvious heterogeneity in one of the studies (Figure 4). After the data from that study were removed, the heterogeneity was significantly reduced, and there were still significant differences in WMD and 95% CI: heterogeneity test, *P*=0.43, *I*^2^ = 0%; WMD = 3.49, 95% CI (1.55, 5.42), *P* < 0.001.

### 3.4. Publication Bias

**Figure 5:**
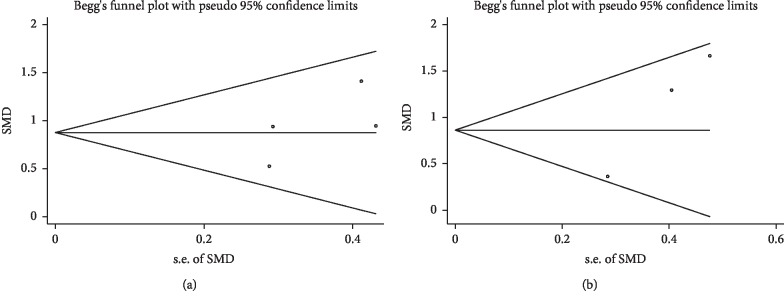
Begg's funnel plot of publication bias in the meta-analysis of the effects of cardiac rehabilitation on peak VO_2_ (a) and LVEF (b) in heart failure patients with CRT.

Egger's test revealed that there was no publication bias in the analyses for peak VO_2_ (*t* = 1.32, *P*=0.318) (Figure 5(a)) and LVEF (*t* = 11.56, *P*=0.055) (Figure 5(b)). The shapes of the funnel plots do not show evidence of asymmetry.

## 4. Discussion

This meta-analysis found that cardiac rehabilitation appears to be safe and effective for high-risk heart failure patients with CRT. The included studies had varying exercise intensity, frequency, and duration. Most studies used moderate-intensity exercise, and the highest intensity was 90% of the peak heart rate. All cardiac rehabilitation programs lasted 2 to 4 months with the frequency of 3–5 sessions/week. Despite the varying exercise intensity, frequency, and duration, the programs were all well tolerated, and the patients did not have any complications from the exercise training. Nevertheless, in cardiac rehabilitation programs, all exercise training programs for heart failure patients should include at least a preliminary assessment with a cardiopulmonary exercise test and cardiac function evaluation [16]. The exercise training may be completely effective only when directly supervised by a physiotherapist or guided by telemonitoring. Appropriate education to promote a healthy and active lifestyle, controlled regular exercise, and motivation to safely increase the exercise load and intensity also play vital roles in the outcomes of heart failure patients [15].

Cardiac rehabilitation is a widely accepted treatment for patients with chronic heart failure [17]. However, cardiac rehabilitation should be considered a comprehensive management strategy. A variety of factors affect the outcome of cardiac rehabilitation, such as patient education, the type of exercise training, the medications prescribed, and psychosocial support [18]. CRT can significantly improve cardiac function in patients with heart failure but has little effect on the peripheral skeletal muscle [19]. However, appropriate and personalized cardiac rehabilitation significantly improves exercise duration, peak oxygen uptake, cardiac function, and peripheral skeletal muscle function [12, 20]. Therefore, in patients with heart failure undergoing cardiac rehabilitation after CRT, the clinical improvement of cardiac function level on the NHYA and quality of life is likely to be explained by the peripheral (muscular and vascular) and cardiac effects of CRT-induced cardiac function enhancement and cardiac rehabilitation exercise [16, 20, 21].

However, other research studies have shown that cardiac rehabilitation (3-4 months) significantly improves the effects of CRT on peak oxygen uptake and cardiac function in patients with heart failure, but prolonging the follow-up time to 12 months does not sustain this effect. Compared with the control group, cardiac rehabilitation failed to result in obviously improved activity tolerance and heart function at 12 months [15]. This result may be affected by the low intensity of home-based exercise training. A higher intensity of training may be required.

This study included a meta-analysis of peak oxygen uptake and LVEF, and the results showed that cardiac rehabilitation can significantly improve peak VO_2_ (WMD 2.17 ml/kg/min, 95% CI 1.42, 2.92) and LVEF (WMD 4.75%, 95% CI 1.53, 7.97) in patients with heart failure undergoing cardiac rehabilitation after CRT and thus may further improve prognosis. The results of this study are consistent with those of most studies on cardiac rehabilitation after CRT, further demonstrating the role of cardiac rehabilitation in patients with heart failure after CRT.

Finally, as CRT is now increasingly used in heart failure patients, the telemonitoring of devices coupled with guided home-based training may be proposed to these patients, either alone or after an initial center-based cardiac rehabilitation program, in order to improve the long-term adherence [16, 22]. So far, there data pertaining to such programs are scarce [15]. Further studies are needed.

There are still some limitations in this study: (1) the number of included studies is not large, and the sample sizes of the included studies are relatively small, meaning that the findings need to be confirmed in large-sample, randomized, double-blind controlled trials; (2) cardiac rehabilitation in China is still in its initial stage, and the standards of cardiac rehabilitation in various research centers are not completely consistent. There are differences in intervention measures, leading to different subsequent influences.

## 5. Conclusions

In conclusion, cardiac rehabilitation can improve exercise capacity and cardiac function in heart failure patients with CRT. Future studies are needed to evaluate whether these beneficial effects of cardiac rehabilitation may translate into an improvement in long-term clinical outcomes among these patients.

## Figures and Tables

**Table 1:** The characteristics of the included studies.

Author	Year	Sample size		Follow-up time (months)	Peak VO_2_ (ml/kg/min, mean ± SD)		LVEF (%)		CR protocol (a: duration, b: frequency, c: exercise mode, d: intensity)	Outcome	Adverse events
		Control	CR		Control	CR	Control	CR			
Patwala et al. [12]	2009	25	25	3	18.07 ± 3.89	20.1 ± 3.84	35.0 ± 7.2	37.3 ± 5.4	(a) 12 weeks (b) 3 sessions/week (c) aerobic exercise, each session consisted of 10 min of treadmill walking, 10 min of cycling, and 10 min of treadmill walking (d) moderate to high intensity, 80% of the peak heart rate achieved in the first 4 weeks, 85% in the next 4 weeks, and 90% in the final 4 weeks	Peak VO_2_, NYHA class, exercise duration, QOL, several other measures of cardiopulmonary fitness, echocardiographic measures	No patients had any adverse events from the exercise training

Belardinelli et al. [13]	2006	10	15	2	15.8 ± 2.1	17.9 ± 2.3	33 ± 6	42 ± 5	(a) 8 weeks (b) 3 sessions/week (c) aerobic exercise, each session consisted of 15 min of stretching exercises, 40 min of cycling, and 5 min of loadless recovery (d) moderate intensity, 60% of the peak VO_2_	Peak VO_2_, functional capacity, QOL, hospital readmission, echocardiographic measures	No patients had any adverse events from the exercise training

Nobre et al. [14]	2016	16	14	4	19.7 ± 1.2	21.6 ± 1.5	29 ± 2	33 ± 4	(a) 4 months (b) 3 sessions/week (c) aerobic exercise, each session consisted of 5 min of stretching exercises, 40 min of treadmill walking, 10 min of local strengthening exercises, and 5 min of cool down with stretching exercises (d) moderate intensity, established by heart rate levels that corresponded to the anaerobic threshold up to 10% below the respiratory compensation point obtained in the cardiopulmonary exercise test	Peak VO_2_, muscle sympathetic nerve activity, exercise duration, echocardiographic measures, forearm blood flow, Ca^2+^ handling gene expression in vastus lateralis muscle	N/A

Smolis-Bak et al. [15]	2015	26	26	3-4/12	13.4 ± 4.2	17.2 ± 3.9			(a) 11 months (b) initial exercise training in the hospital setting for 3 weeks on average, then at home with telemonitoring for 5 sessions/week (c) aerobic exercise, active exercises of small and subsequently larger muscle groups of the lower and upper limbs, respiratory exercises, range-of-motion exercises of the shoulder joint on the implantation side (d) low intensity	Peak VO_2_, peak VCO_2_, treadmill test duration, echocardiographic measures, QOL	No significant differences in ICD interventions, mortality, or hospitalization rates between the groups

CR: cardiac rehabilitation; peak VO_2_: peak oxygen uptake; peak VCO_2_: peak carbon dioxide elimination; LVEF: left ventricular ejection fraction; QOL: quality of life; N/A: not available; ICD: implantable cardioverter-defibrillator.

## Data Availability

The data supporting this meta-analysis are from previously reported studies and datasets, which have been cited. The processed data are available in the manuscript.
